# Aqua­bis­(4-chloro­benz­yl)bis­(nicotinato-κ^2^ *O*,*O*′)tin(IV)

**DOI:** 10.1107/S1600536811015728

**Published:** 2011-05-07

**Authors:** Thy Chun Keng, Kong Mun Lo, Seik Weng Ng

**Affiliations:** a Department of Chemistry, University of Malaya, 50603 Kuala Lumpur, Malaysia

## Abstract

In the title mol­ecule, [Sn(C_7_H_6_Cl)_2_(C_6_H_4_NO_2_)_2_(H_2_O)], the O atoms of the two chelating nicotinate groups and the O atom of the coordinated water mol­ecule comprise the penta­gonal plane of the *trans*-C_2_SnO_5_ penta­gonal–bipyramid [C—Sn—C = 178.62 (11) °] surrounding the Sn^IV^ atom. In the crystal, adjacent mol­ecules are linked by O—H⋯N hydrogen bonds, generating a chain running along the body diagonal of the triclinic unit cell.

## Related literature

For the direct synthesis of the organotin chloride reactant, see: Sisido *et al.* (1961 ▶). For the dinuclear bromo analog, see: Keng *et al.* (2010 ▶). For a review of the crystal structures of organotin carboxyl­ates, see: Tiekink (1991 ▶, 1994 ▶).
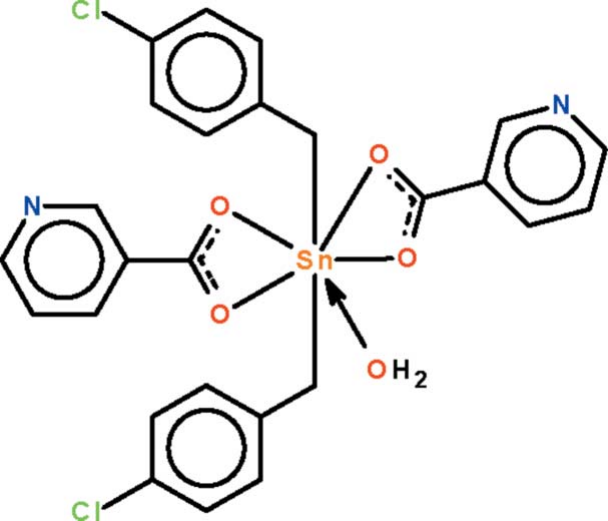


## Experimental

### 

#### Crystal data

[Sn(C_7_H_6_Cl)_2_(C_6_H_4_NO_2_)_2_(H_2_O)]
*M* *_r_* = 632.05
Triclinic, 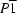

*a* = 9.0219 (1) Å
*b* = 10.5929 (1) Å
*c* = 14.5866 (2) Å
α = 79.6490 (5)°
β = 87.6290 (5)°
γ = 66.5051 (4)°
*V* = 1256.93 (3) Å^3^
*Z* = 2
Mo *K*α radiation
μ = 1.27 mm^−1^
*T* = 100 K
0.35 × 0.30 × 0.25 mm

#### Data collection

Bruker SMART APEX diffractometer
Absorption correction: multi-scan (*SADABS*; Sheldrick, 1996 ▶) *T*_min_ = 0.665, *T*_max_ = 0.742
11404 measured reflections
5673 independent reflections
5416 reflections with *I* > 2σ(*I*)
*R*_int_ = 0.022

#### Refinement

*R*[*F* ^2^ > 2σ(*F* ^2^)] = 0.039
*wR*(*F* ^2^) = 0.133
*S* = 1.07
5673 reflections
326 parameters
H-atom parameters constrained
Δρ_max_ = 2.51 e Å^−3^
Δρ_min_ = −1.97 e Å^−3^

### 

Data collection: *APEX2* (Bruker, 2009 ▶); cell refinement: *SAINT* (Bruker, 2009 ▶); data reduction: *SAINT*; program(s) used to solve structure: *SHELXS97* (Sheldrick, 2008 ▶); program(s) used to refine structure: *SHELXL97* (Sheldrick, 2008 ▶); molecular graphics: *X-SEED* (Barbour, 2001 ▶); software used to prepare material for publication: *publCIF* (Westrip, 2010 ▶).

## Supplementary Material

Crystal structure: contains datablocks global, I. DOI: 10.1107/S1600536811015728/bt5527sup1.cif

Structure factors: contains datablocks I. DOI: 10.1107/S1600536811015728/bt5527Isup2.hkl

Additional supplementary materials:  crystallographic information; 3D view; checkCIF report

## Figures and Tables

**Table 1:** Hydrogen-bond geometry (Å, °)

*D*—H⋯*A*	*D*—H	H⋯*A*	*D*⋯*A*	*D*—H⋯*A*
O1w—H1⋯N1^i^	0.84	1.93	2.721 (3)	158
O1w—H2⋯N2^ii^	0.84	2.01	2.754 (3)	146

Symmetry codes: (i) 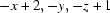; (ii) 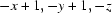.

**Fig. 1.**
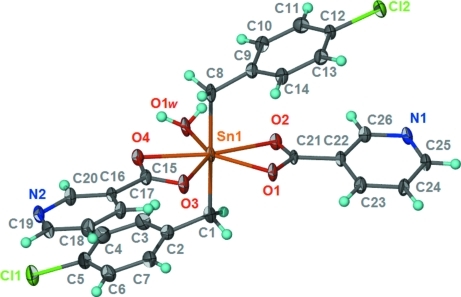
Thermal ellipsoid plot (Barbour, 2001) of Sn(H2O)(C7H6Cl)2(C6H4NO2)2 at the 70% probability level; hydrogen atoms are drawn as spheres of arbitrary radius.

**Fig. 2.**
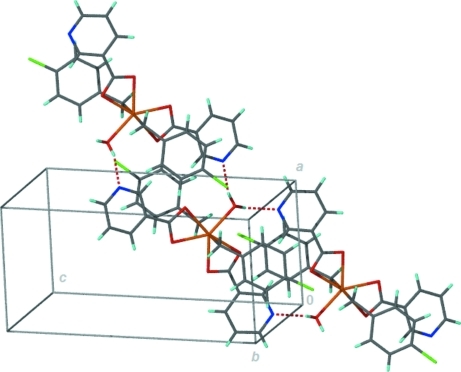
Packing diagram.

## supplementary crystallographic information

### Comment

Nicotinic acid affords a large number of compounds with organotins. For the diorganotin system in particular, the nicotinate ion can behave as an *O*,*O*'-chelate, but when two ions bind to a diorganotin cation, there is some space in the coordination polyhedron to admit a small ligand such as a water molecule (Tiekink, 1991; 1994). The bromo analog of the title compound (Scheme I) exists as a dinuclear compound as the N atom engages in coordination (Keng *et al.*, 2010). The O atoms of the two chelating nicotinate groups and the O atom of the coordinated water molecule comprise the pentagonal plane of the *trans*-C_2_SnO5 pentagonal-bipyramid [C–Sn–C 178.6 (1) °] surrounding the Sn^IV^ atom in title compound (Fig. 1). The N atom does not engage in binding to an adjacent metal center. Instead, both N atoms serve as hydrogen bond acceptors (Table 1). Adjacent molecules are linked by O–H···N hydrogen bonds to generate a chain along [1 - 1 1].

### Experimental

Di(4-chlorobenzyl)tin oxide was prepared by the base hydrolysis of di(4-chlorobenzyl)tin dichloride with 10% sodium hydroxide. The diorganotin dichloride was synthesized by the direct reaction of 4-chlorobenzyl chloride and metallic tin according to a literature procedure (Sisido *et al.*, 1961). The diorganotin oxide (0.39 g, 1 mmol) and nicotinic acid (0.25 g, 2 mmol) were heated in ethanol (100 ml) for an hour until the oxide dissolved. The solution was filtered; slow evaporation of the filtrate gave colorless crystals.

### Refinement

Carbon-bound H-atoms were placed in calculated positions (C—H 0.95 to 0.99, O–H 0.84 Å) and were included in the refinement in the riding model approximation, with *U*(H) set to 1.2–1.5 times *U*_eq_(C,*O*).

The final difference Fourier map had a peak in the vicinity of Sn1 as well as a hole in the vicinity of the same atom. The peaks/holes affected the the weighting scheme, which had a somewhat large value as the first parameter but a small value for the second parameter. The weighting scheme could be marginally improved by lowering the 2θ limit to 50 °.

### Crystal data

**Table:**

[Sn(C_7_H_6_Cl)_2_(C_6_H_4_NO_2_)_2_(H_2_O)]	*Z* = 2
*M**_r_* = 632.05	*F*(000) = 632
Triclinic, *P*1	*D*_x_ = 1.670 Mg m^−^^3^
Hall symbol: -P 1	Mo *K*α radiation, λ = 0.71073 Å
*a* = 9.0219 (1) Å	Cell parameters from 9937 reflections
*b* = 10.5929 (1) Å	θ = 2.5–28.4°
*c* = 14.5866 (2) Å	µ = 1.27 mm^−^^1^
α = 79.6490 (5)°	*T* = 100 K
β = 87.6290 (5)°	Block, colorless
γ = 66.5051 (4)°	0.35 × 0.30 × 0.25 mm
*V* = 1256.93 (3) Å^3^	

### Data collection

**Table:**

Bruker SMART APEX diffractometer	5673 independent reflections
Radiation source: fine-focus sealed tube	5416 reflections with *I* > 2σ(*I*)
graphite	*R*_int_ = 0.022
ω scans	θ_max_ = 27.5°, θ_min_ = 2.3°
Absorption correction: multi-scan (*SADABS*; Sheldrick, 1996)	*h* = −11→11
*T*_min_ = 0.665, *T*_max_ = 0.742	*k* = −13→13
11404 measured reflections	*l* = −18→18

### Refinement

**Table:**

Refinement on *F*^2^	Primary atom site location: structure-invariant direct methods
Least-squares matrix: full	Secondary atom site location: difference Fourier map
*R*[*F*^2^ > 2σ(*F*^2^)] = 0.039	Hydrogen site location: inferred from neighbouring sites
*wR*(*F*^2^) = 0.133	H-atom parameters constrained
*S* = 1.07	*w* = 1/[σ^2^(*F*_o_^2^) + (0.1028*P*)^2^ + 1.3179*P*] where *P* = (*F*_o_^2^ + 2*F*_c_^2^)/3
5673 reflections	(Δ/σ)_max_ = 0.001
326 parameters	Δρ_max_ = 2.51 e Å^−^^3^
0 restraints	Δρ_min_ = −1.96 e Å^−^^3^

### Fractional atomic coordinates and isotropic or equivalent isotropic displacement parameters (Å^2^)

**Table:**

	*x*	*y*	*z*	*U*_iso_*/*U*_eq_	
Sn1	0.63205 (2)	0.308637 (17)	0.300723 (11)	0.01463 (11)	
Cl1	0.23192 (13)	0.32379 (11)	−0.10807 (6)	0.0365 (2)	
Cl2	1.16041 (10)	0.23720 (10)	0.67438 (6)	0.02660 (19)	
O1	0.5519 (3)	0.3053 (2)	0.45081 (15)	0.0183 (4)	
O2	0.7813 (3)	0.1357 (2)	0.42515 (15)	0.0172 (4)	
O3	0.3946 (3)	0.5014 (2)	0.27412 (15)	0.0198 (4)	
O4	0.5422 (3)	0.4480 (2)	0.15155 (15)	0.0170 (4)	
O1W	0.8289 (3)	0.1801 (2)	0.21474 (15)	0.0202 (4)	
H1	0.9173	0.1454	0.2452	0.030*	
H2	0.8364	0.2314	0.1653	0.030*	
N1	0.8693 (3)	−0.0102 (3)	0.71325 (18)	0.0180 (5)	
N2	0.1562 (3)	0.7425 (3)	−0.02511 (18)	0.0191 (5)	
C1	0.5052 (4)	0.1792 (4)	0.2843 (2)	0.0213 (6)	
H1A	0.5814	0.0799	0.2990	0.026*	
H1B	0.4182	0.1941	0.3299	0.026*	
C2	0.4330 (4)	0.2070 (3)	0.1894 (2)	0.0195 (6)	
C3	0.5243 (4)	0.1420 (4)	0.1180 (2)	0.0225 (6)	
H3	0.6308	0.0730	0.1323	0.027*	
C4	0.4630 (4)	0.1761 (4)	0.0277 (2)	0.0268 (7)	
H4	0.5276	0.1323	−0.0199	0.032*	
C5	0.3059 (5)	0.2749 (4)	0.0068 (2)	0.0251 (7)	
C6	0.2098 (4)	0.3362 (4)	0.0761 (3)	0.0247 (7)	
H6	0.1012	0.4009	0.0619	0.030*	
C7	0.2735 (4)	0.3024 (3)	0.1670 (2)	0.0207 (6)	
H7	0.2074	0.3448	0.2146	0.025*	
C8	0.7636 (4)	0.4347 (3)	0.3155 (2)	0.0204 (6)	
H8A	0.8347	0.4327	0.2618	0.025*	
H8B	0.6853	0.5327	0.3126	0.025*	
C9	0.8648 (4)	0.3911 (3)	0.4032 (2)	0.0176 (6)	
C10	1.0255 (4)	0.2947 (3)	0.4075 (2)	0.0188 (6)	
H10	1.0731	0.2611	0.3526	0.023*	
C11	1.1167 (4)	0.2473 (3)	0.4901 (2)	0.0205 (6)	
H11	1.2255	0.1808	0.4923	0.025*	
C12	1.0476 (4)	0.2978 (3)	0.5691 (2)	0.0180 (6)	
C13	0.8903 (4)	0.3960 (3)	0.5674 (2)	0.0190 (6)	
H13	0.8452	0.4316	0.6221	0.023*	
C14	0.7993 (4)	0.4417 (3)	0.4843 (2)	0.0184 (6)	
H14	0.6907	0.5084	0.4826	0.022*	
C15	0.4151 (4)	0.5225 (3)	0.1867 (2)	0.0162 (5)	
C16	0.2813 (3)	0.6374 (3)	0.1270 (2)	0.0150 (5)	
C17	0.1543 (4)	0.7338 (3)	0.1676 (2)	0.0180 (6)	
H17	0.1538	0.7305	0.2332	0.022*	
C18	0.0282 (4)	0.8350 (3)	0.1102 (2)	0.0208 (6)	
H18	−0.0597	0.9035	0.1353	0.025*	
C19	0.0336 (4)	0.8336 (3)	0.0151 (2)	0.0197 (6)	
H19	−0.0545	0.9010	−0.0237	0.024*	
C20	0.2787 (4)	0.6464 (3)	0.0306 (2)	0.0192 (6)	
H20	0.3673	0.5818	0.0032	0.023*	
C21	0.6771 (3)	0.1968 (3)	0.48055 (19)	0.0141 (5)	
C22	0.6982 (4)	0.1403 (3)	0.5829 (2)	0.0155 (5)	
C23	0.5673 (4)	0.1831 (3)	0.6405 (2)	0.0191 (6)	
H23	0.4650	0.2506	0.6157	0.023*	
C24	0.5882 (4)	0.1257 (4)	0.7348 (2)	0.0193 (6)	
H24	0.5006	0.1519	0.7756	0.023*	
C25	0.7402 (4)	0.0293 (3)	0.7676 (2)	0.0191 (6)	
H25	0.7543	−0.0112	0.8319	0.023*	
C26	0.8467 (4)	0.0453 (3)	0.6216 (2)	0.0166 (5)	
H26	0.9363	0.0181	0.5822	0.020*	

### Atomic displacement parameters (Å^2^)

**Table:**

	*U*^11^	*U*^22^	*U*^33^	*U*^12^	*U*^13^	*U*^23^
Sn1	0.01514 (15)	0.01586 (15)	0.00806 (15)	−0.00249 (10)	−0.00275 (9)	0.00174 (9)
Cl1	0.0496 (5)	0.0521 (6)	0.0153 (4)	−0.0313 (5)	−0.0098 (4)	0.0043 (4)
Cl2	0.0204 (4)	0.0416 (5)	0.0149 (4)	−0.0118 (3)	−0.0048 (3)	0.0020 (3)
O1	0.0197 (10)	0.0194 (10)	0.0089 (10)	−0.0026 (8)	−0.0024 (7)	0.0030 (8)
O2	0.0180 (10)	0.0174 (10)	0.0124 (10)	−0.0041 (8)	−0.0009 (8)	−0.0004 (8)
O3	0.0190 (10)	0.0217 (10)	0.0101 (10)	−0.0008 (8)	−0.0013 (8)	0.0017 (8)
O4	0.0175 (10)	0.0173 (10)	0.0122 (10)	−0.0039 (8)	−0.0021 (8)	0.0005 (8)
O1W	0.0174 (10)	0.0237 (11)	0.0095 (9)	0.0003 (8)	−0.0043 (8)	0.0028 (8)
N1	0.0192 (12)	0.0180 (11)	0.0116 (12)	−0.0035 (9)	−0.0051 (9)	0.0022 (9)
N2	0.0208 (12)	0.0206 (12)	0.0119 (12)	−0.0060 (10)	−0.0045 (9)	0.0029 (10)
C1	0.0223 (15)	0.0242 (15)	0.0148 (15)	−0.0090 (13)	−0.0055 (12)	0.0038 (12)
C2	0.0222 (15)	0.0215 (14)	0.0161 (15)	−0.0115 (12)	−0.0025 (11)	0.0009 (11)
C3	0.0233 (15)	0.0241 (15)	0.0204 (16)	−0.0100 (12)	−0.0018 (12)	−0.0027 (12)
C4	0.0326 (17)	0.0342 (18)	0.0211 (16)	−0.0198 (15)	0.0022 (13)	−0.0078 (14)
C5	0.0357 (18)	0.0303 (17)	0.0152 (15)	−0.0216 (15)	−0.0060 (13)	0.0025 (13)
C6	0.0237 (15)	0.0241 (15)	0.0233 (17)	−0.0096 (13)	−0.0088 (13)	0.0054 (13)
C7	0.0215 (14)	0.0224 (14)	0.0169 (15)	−0.0089 (12)	−0.0023 (11)	0.0008 (12)
C8	0.0264 (16)	0.0196 (14)	0.0111 (14)	−0.0074 (12)	−0.0052 (12)	0.0047 (11)
C9	0.0229 (14)	0.0177 (13)	0.0127 (14)	−0.0097 (12)	−0.0026 (11)	0.0008 (11)
C10	0.0213 (14)	0.0202 (14)	0.0146 (14)	−0.0084 (12)	0.0018 (11)	−0.0027 (11)
C11	0.0186 (14)	0.0212 (14)	0.0208 (15)	−0.0069 (11)	0.0009 (11)	−0.0035 (12)
C12	0.0189 (14)	0.0222 (14)	0.0122 (14)	−0.0091 (12)	−0.0035 (11)	0.0020 (11)
C13	0.0226 (15)	0.0202 (14)	0.0151 (14)	−0.0097 (12)	0.0010 (11)	−0.0027 (11)
C14	0.0209 (14)	0.0168 (13)	0.0146 (14)	−0.0060 (11)	−0.0023 (11)	0.0012 (11)
C15	0.0193 (13)	0.0154 (13)	0.0121 (13)	−0.0063 (11)	−0.0026 (10)	0.0013 (10)
C16	0.0156 (13)	0.0168 (13)	0.0103 (13)	−0.0058 (11)	−0.0030 (10)	0.0030 (10)
C17	0.0197 (13)	0.0180 (13)	0.0115 (13)	−0.0043 (11)	−0.0017 (10)	0.0022 (11)
C18	0.0193 (14)	0.0182 (13)	0.0172 (15)	−0.0014 (11)	−0.0005 (11)	0.0014 (11)
C19	0.0178 (13)	0.0182 (13)	0.0165 (15)	−0.0030 (11)	−0.0071 (11)	0.0051 (11)
C20	0.0215 (14)	0.0199 (14)	0.0117 (14)	−0.0048 (11)	−0.0014 (11)	0.0004 (11)
C21	0.0151 (12)	0.0152 (12)	0.0081 (13)	−0.0044 (10)	−0.0031 (10)	0.0043 (10)
C22	0.0196 (13)	0.0168 (13)	0.0091 (13)	−0.0072 (11)	−0.0017 (10)	0.0005 (10)
C23	0.0178 (13)	0.0201 (14)	0.0138 (14)	−0.0039 (11)	−0.0019 (10)	0.0022 (11)
C24	0.0210 (15)	0.0252 (15)	0.0093 (14)	−0.0080 (12)	0.0018 (11)	−0.0002 (11)
C25	0.0225 (14)	0.0216 (14)	0.0106 (13)	−0.0081 (12)	−0.0037 (11)	0.0028 (11)
C26	0.0183 (13)	0.0154 (12)	0.0135 (13)	−0.0047 (11)	−0.0012 (10)	−0.0003 (10)

### Geometric parameters (Å, °)

**Table:**

Sn1—C8	2.151 (3)	C7—H7	0.9500
Sn1—C1	2.153 (3)	C8—C9	1.494 (4)
Sn1—O1W	2.254 (2)	C8—H8A	0.9900
Sn1—O1	2.276 (2)	C8—H8B	0.9900
Sn1—O3	2.281 (2)	C9—C14	1.398 (4)
Sn1—O2	2.346 (2)	C9—C10	1.397 (4)
Sn1—O4	2.368 (2)	C10—C11	1.385 (4)
Sn1—C21	2.653 (3)	C10—H10	0.9500
Sn1—C15	2.666 (3)	C11—C12	1.378 (4)
Cl1—C5	1.739 (3)	C11—H11	0.9500
Cl2—C12	1.749 (3)	C12—C13	1.383 (4)
O1—C21	1.269 (4)	C13—C14	1.390 (4)
O2—C21	1.264 (4)	C13—H13	0.9500
O3—C15	1.273 (4)	C14—H14	0.9500
O4—C15	1.255 (4)	C15—C16	1.494 (4)
O1W—H1	0.8400	C16—C17	1.391 (4)
O1W—H2	0.8400	C16—C20	1.393 (4)
N1—C25	1.347 (4)	C17—C18	1.389 (4)
N1—C26	1.349 (4)	C17—H17	0.9500
N2—C19	1.340 (4)	C18—C19	1.388 (4)
N2—C20	1.341 (4)	C18—H18	0.9500
C1—C2	1.483 (4)	C19—H19	0.9500
C1—H1A	0.9900	C20—H20	0.9500
C1—H1B	0.9900	C21—C22	1.496 (4)
C2—C7	1.400 (4)	C22—C26	1.382 (4)
C2—C3	1.404 (5)	C22—C23	1.391 (4)
C3—C4	1.380 (5)	C23—C24	1.387 (4)
C3—H3	0.9500	C23—H23	0.9500
C4—C5	1.392 (5)	C24—C25	1.384 (4)
C4—H4	0.9500	C24—H24	0.9500
C5—C6	1.381 (5)	C25—H25	0.9500
C6—C7	1.395 (5)	C26—H26	0.9500
C6—H6	0.9500		
			
C8—Sn1—C1	178.62 (11)	C2—C7—H7	119.4
C8—Sn1—O1W	90.93 (11)	C9—C8—Sn1	115.3 (2)
C1—Sn1—O1W	87.70 (11)	C9—C8—H8A	108.5
C8—Sn1—O1	92.45 (10)	Sn1—C8—H8A	108.5
C1—Sn1—O1	88.51 (11)	C9—C8—H8B	108.5
O1W—Sn1—O1	140.24 (8)	Sn1—C8—H8B	108.5
C8—Sn1—O3	91.45 (11)	H8A—C8—H8B	107.5
C1—Sn1—O3	89.65 (11)	C14—C9—C10	118.2 (3)
O1W—Sn1—O3	137.17 (8)	C14—C9—C8	120.8 (3)
O1—Sn1—O3	82.34 (8)	C10—C9—C8	121.0 (3)
C8—Sn1—O2	91.61 (10)	C11—C10—C9	121.2 (3)
C1—Sn1—O2	88.10 (10)	C11—C10—H10	119.4
O1W—Sn1—O2	83.33 (8)	C9—C10—H10	119.4
O1—Sn1—O2	56.98 (8)	C12—C11—C10	119.1 (3)
O3—Sn1—O2	139.30 (8)	C12—C11—H11	120.5
C8—Sn1—O4	87.44 (10)	C10—C11—H11	120.5
C1—Sn1—O4	92.48 (10)	C11—C12—C13	121.5 (3)
O1W—Sn1—O4	80.87 (8)	C11—C12—Cl2	119.6 (2)
O1—Sn1—O4	138.86 (8)	C13—C12—Cl2	118.8 (2)
O3—Sn1—O4	56.55 (8)	C12—C13—C14	118.9 (3)
O2—Sn1—O4	164.15 (8)	C12—C13—H13	120.6
C8—Sn1—C21	91.62 (10)	C14—C13—H13	120.6
C1—Sn1—C21	88.76 (11)	C13—C14—C9	121.1 (3)
O1W—Sn1—C21	111.77 (8)	C13—C14—H14	119.5
O1—Sn1—C21	28.55 (8)	C9—C14—H14	119.5
O3—Sn1—C21	110.90 (8)	O4—C15—O3	121.3 (3)
O2—Sn1—C21	28.44 (8)	O4—C15—C16	121.0 (3)
O4—Sn1—C21	167.35 (9)	O3—C15—C16	117.7 (3)
C8—Sn1—C15	90.37 (10)	O4—C15—Sn1	62.65 (16)
C1—Sn1—C15	90.20 (11)	O3—C15—Sn1	58.73 (15)
O1W—Sn1—C15	108.75 (8)	C16—C15—Sn1	174.4 (2)
O1—Sn1—C15	110.83 (8)	C17—C16—C20	119.1 (3)
O3—Sn1—C15	28.49 (8)	C17—C16—C15	120.2 (3)
O2—Sn1—C15	167.73 (9)	C20—C16—C15	120.6 (3)
O4—Sn1—C15	28.09 (9)	C18—C17—C16	118.5 (3)
C21—Sn1—C15	139.38 (9)	C18—C17—H17	120.8
C21—O1—Sn1	92.46 (17)	C16—C17—H17	120.8
C21—O2—Sn1	89.38 (17)	C19—C18—C17	118.4 (3)
C15—O3—Sn1	92.78 (18)	C19—C18—H18	120.8
C15—O4—Sn1	89.26 (18)	C17—C18—H18	120.8
Sn1—O1W—H1	109.5	N2—C19—C18	123.8 (3)
Sn1—O1W—H2	109.5	N2—C19—H19	118.1
H1—O1W—H2	109.5	C18—C19—H19	118.1
C25—N1—C26	117.6 (3)	N2—C20—C16	122.7 (3)
C19—N2—C20	117.5 (3)	N2—C20—H20	118.7
C2—C1—Sn1	113.9 (2)	C16—C20—H20	118.7
C2—C1—H1A	108.8	O2—C21—O1	121.1 (3)
Sn1—C1—H1A	108.8	O2—C21—C22	120.1 (3)
C2—C1—H1B	108.8	O1—C21—C22	118.7 (3)
Sn1—C1—H1B	108.8	O2—C21—Sn1	62.18 (15)
H1A—C1—H1B	107.7	O1—C21—Sn1	58.99 (14)
C7—C2—C3	117.6 (3)	C22—C21—Sn1	177.4 (2)
C7—C2—C1	121.3 (3)	C26—C22—C23	119.1 (3)
C3—C2—C1	121.0 (3)	C26—C22—C21	120.9 (3)
C4—C3—C2	121.5 (3)	C23—C22—C21	120.0 (3)
C4—C3—H3	119.2	C24—C23—C22	119.0 (3)
C2—C3—H3	119.2	C24—C23—H23	120.5
C3—C4—C5	119.5 (3)	C22—C23—H23	120.5
C3—C4—H4	120.3	C25—C24—C23	118.2 (3)
C5—C4—H4	120.3	C25—C24—H24	120.9
C6—C5—C4	120.6 (3)	C23—C24—H24	120.9
C6—C5—Cl1	120.0 (3)	N1—C25—C24	123.5 (3)
C4—C5—Cl1	119.5 (3)	N1—C25—H25	118.2
C5—C6—C7	119.5 (3)	C24—C25—H25	118.2
C5—C6—H6	120.3	N1—C26—C22	122.5 (3)
C7—C6—H6	120.3	N1—C26—H26	118.8
C6—C7—C2	121.2 (3)	C22—C26—H26	118.8
C6—C7—H7	119.4		
			
C8—Sn1—O1—C21	−88.89 (19)	C12—C13—C14—C9	−0.7 (5)
C1—Sn1—O1—C21	90.13 (19)	C10—C9—C14—C13	−1.0 (4)
O1W—Sn1—O1—C21	5.5 (2)	C8—C9—C14—C13	176.6 (3)
O3—Sn1—O1—C21	179.97 (18)	Sn1—O4—C15—O3	3.8 (3)
O2—Sn1—O1—C21	1.43 (16)	Sn1—O4—C15—C16	−175.1 (2)
O4—Sn1—O1—C21	−177.81 (15)	Sn1—O3—C15—O4	−3.9 (3)
C15—Sn1—O1—C21	179.78 (17)	Sn1—O3—C15—C16	175.0 (2)
C8—Sn1—O2—C21	90.45 (19)	C8—Sn1—C15—O4	83.86 (19)
C1—Sn1—O2—C21	−90.90 (19)	C1—Sn1—C15—O4	−94.88 (19)
O1W—Sn1—O2—C21	−178.80 (18)	O1W—Sn1—C15—O4	−7.25 (19)
O1—Sn1—O2—C21	−1.44 (16)	O1—Sn1—C15—O4	176.63 (16)
O3—Sn1—O2—C21	−3.7 (2)	O3—Sn1—C15—O4	176.2 (3)
O4—Sn1—O2—C21	176.7 (2)	O2—Sn1—C15—O4	−176.8 (3)
C15—Sn1—O2—C21	−8.7 (5)	C21—Sn1—C15—O4	176.79 (15)
C8—Sn1—O3—C15	88.09 (19)	C8—Sn1—C15—O3	−92.37 (19)
C1—Sn1—O3—C15	−91.08 (19)	C1—Sn1—C15—O3	88.88 (19)
O1W—Sn1—O3—C15	−4.9 (2)	O1W—Sn1—C15—O3	176.52 (17)
O1—Sn1—O3—C15	−179.63 (18)	O1—Sn1—C15—O3	0.40 (19)
O2—Sn1—O3—C15	−177.75 (15)	O2—Sn1—C15—O3	6.9 (5)
O4—Sn1—O3—C15	2.13 (16)	O4—Sn1—C15—O3	−176.2 (3)
C21—Sn1—O3—C15	−179.61 (17)	C21—Sn1—C15—O3	0.6 (2)
C8—Sn1—O4—C15	−95.59 (19)	O4—C15—C16—C17	−169.0 (3)
C1—Sn1—O4—C15	85.78 (19)	O3—C15—C16—C17	12.1 (4)
O1W—Sn1—O4—C15	173.05 (18)	O4—C15—C16—C20	13.6 (4)
O1—Sn1—O4—C15	−4.8 (2)	O3—C15—C16—C20	−165.4 (3)
O3—Sn1—O4—C15	−2.15 (16)	C20—C16—C17—C18	0.6 (4)
O2—Sn1—O4—C15	177.5 (2)	C15—C16—C17—C18	−176.9 (3)
C21—Sn1—O4—C15	−9.6 (4)	C16—C17—C18—C19	1.1 (4)
O1W—Sn1—C1—C2	−73.1 (2)	C20—N2—C19—C18	1.0 (5)
O1—Sn1—C1—C2	146.5 (2)	C17—C18—C19—N2	−2.0 (5)
O3—Sn1—C1—C2	64.2 (2)	C19—N2—C20—C16	0.9 (5)
O2—Sn1—C1—C2	−156.5 (2)	C17—C16—C20—N2	−1.7 (4)
O4—Sn1—C1—C2	7.7 (2)	C15—C16—C20—N2	175.8 (3)
C21—Sn1—C1—C2	175.1 (2)	Sn1—O2—C21—O1	2.5 (3)
C15—Sn1—C1—C2	35.7 (2)	Sn1—O2—C21—C22	−178.6 (2)
Sn1—C1—C2—C7	−92.4 (3)	Sn1—O1—C21—O2	−2.6 (3)
Sn1—C1—C2—C3	85.4 (3)	Sn1—O1—C21—C22	178.5 (2)
C7—C2—C3—C4	3.5 (5)	C8—Sn1—C21—O2	−90.37 (18)
C1—C2—C3—C4	−174.3 (3)	C1—Sn1—C21—O2	88.30 (19)
C2—C3—C4—C5	−1.4 (5)	O1W—Sn1—C21—O2	1.28 (19)
C3—C4—C5—C6	−1.7 (5)	O1—Sn1—C21—O2	177.5 (3)
C3—C4—C5—Cl1	177.0 (3)	O3—Sn1—C21—O2	177.45 (16)
C4—C5—C6—C7	2.5 (5)	O4—Sn1—C21—O2	−175.9 (3)
Cl1—C5—C6—C7	−176.2 (2)	C15—Sn1—C21—O2	177.16 (15)
C5—C6—C7—C2	−0.2 (5)	C8—Sn1—C21—O1	92.15 (19)
C3—C2—C7—C6	−2.7 (5)	C1—Sn1—C21—O1	−89.18 (19)
C1—C2—C7—C6	175.1 (3)	O1W—Sn1—C21—O1	−176.20 (16)
O1W—Sn1—C8—C9	−95.9 (2)	O3—Sn1—C21—O1	−0.03 (19)
O1—Sn1—C8—C9	44.4 (2)	O2—Sn1—C21—O1	−177.5 (3)
O3—Sn1—C8—C9	126.8 (2)	O4—Sn1—C21—O1	6.6 (5)
O2—Sn1—C8—C9	−12.6 (2)	C15—Sn1—C21—O1	−0.3 (2)
O4—Sn1—C8—C9	−176.8 (2)	O2—C21—C22—C26	16.9 (4)
C21—Sn1—C8—C9	15.9 (2)	O1—C21—C22—C26	−164.2 (3)
C15—Sn1—C8—C9	155.3 (2)	O2—C21—C22—C23	−163.0 (3)
Sn1—C8—C9—C14	−87.8 (3)	O1—C21—C22—C23	15.9 (4)
Sn1—C8—C9—C10	89.7 (3)	C26—C22—C23—C24	−2.1 (4)
C14—C9—C10—C11	1.7 (4)	C21—C22—C23—C24	177.8 (3)
C8—C9—C10—C11	−175.9 (3)	C22—C23—C24—C25	0.9 (5)
C9—C10—C11—C12	−0.8 (5)	C26—N1—C25—C24	−1.7 (5)
C10—C11—C12—C13	−0.9 (5)	C23—C24—C25—N1	1.0 (5)
C10—C11—C12—Cl2	179.2 (2)	C25—N1—C26—C22	0.4 (4)
C11—C12—C13—C14	1.6 (5)	C23—C22—C26—N1	1.4 (4)
Cl2—C12—C13—C14	−178.5 (2)	C21—C22—C26—N1	−178.4 (3)

### Hydrogen-bond geometry (Å, °)

**Table:**

*D*—H···*A*	*D*—H	H···*A*	*D*···*A*	*D*—H···*A*
O1w—H1···N1^i^	0.84	1.93	2.721 (3)	158
O1w—H2···N2^ii^	0.84	2.01	2.754 (3)	146

Symmetry codes: (i) −*x*+2, −*y*, −*z*+1; (ii) −*x*+1, −*y*+1, −*z*.
